# Deutsches Register www.Covid19-Rheuma.de: Statusbericht nach 1 Jahr der Pandemie

**DOI:** 10.1007/s00393-021-01034-y

**Published:** 2021-07-01

**Authors:** Rebecca Hasseli, Alexander Pfeil, Bimba Franziska Hoyer, Hanns-Martin Lorenz, Anne C. Regierer, Jutta G. Richter, Tim Schmeiser, Anja Strangfeld, Reinhard E. Voll, Andreas Krause, Hendrik Schulze-Koops, Ulf Müller-Ladner, Christof Specker

**Affiliations:** 1 Abteilung für Rheumatologie, Klinische Immunologie, Osteologie und Physikalische Medizin, Justus-Liebig-Universität Gießen, Campus Kerckhoff, Benekestr. 2–8, 61231 Bad Nauheim, Deutschland; 2 Funktionsbereich Rheumatologie und Osteologie, Klinik für Innere Medizin III, Universitätsklinikum Jena, Jena, Deutschland; 3 Sektion für Rheumatologie, 1. Medizinische Klinik, Universitätskrankenhaus Schleswig-Holstein Campus Kiel, Kiel, Deutschland; 4 Sektion Rheumatologie, Medizinische Klinik V, Universitätsklinikum Heidelberg, Heidelberg, Deutschland; 5 Programmbereich Epidemiologie und Versorgungsforschung, Deutsches Rheuma-Forschungszentrum, Berlin, Deutschland; 6 Poliklinik, Funktionsbereich und Hiller Forschungszentrum für Rheumatologie, Universitätsklinikum Düsseldorf, Heinrich-Heine-Universität Düsseldorf, Düsseldorf, Deutschland; 7 Rheumatologie im Veedel, Köln, Deutschland; 8 Klinik für Rheumatologie und Klinische Immunologie, Universitätsklinikum Freiburg, Medizinische Fakultät, Universität Freiburg, Freiburg, Deutschland; 9 Abteilung für Rheumatologie, Osteologie und Klinische Immunologie, Immanuel Krankenhaus Berlin, Berlin, Deutschland; 10 Sektion Rheumatologie und Klinische Immunologie, Medizinische Klinik und Poliklinik IV, Ludwig-Maximilians-Universität München, München, Deutschland; 11 Klinik für Rheumatologie & Klinische Immunologie, Kliniken Essen-Mitte, Essen, Deutschland

**Keywords:** Entzündlich-rheumatische Erkrankungen, Immunmodulation, SARS-CoV‑2, Glukokortikoide, Risikofaktoren, Inflammatory rheumatic diseases, Immunomodulation, SARS-CoV‑2, Glucocorticoids, Risk factors

## Abstract

Durch das COVID-19-Register (www.covid19-rheuma.de) der Deutschen Gesellschaft für Rheumatologie erfolgte erstmalig die Erfassung und systematische Evaluation einer viralen Infektion bei Patienten mit entzündlich rheumatischen Erkrankungen (ERE). Hierdurch war und ist eine schnelle Generierung von wissenschaftlichen Daten möglich, welche helfen, die Betreuung von Patienten mit ERE im Rahmen der Pandemie zu verbessern. Neben der Bestätigung allgemeiner Risikofaktoren – auch für Patienten mit ERE – wie Patientenalter und Komorbiditäten (z. B. kardiovaskuläre, chronische Lungen- und Nierenerkrankungen) konnten die Einnahme von Glukokortikoiden und die Krankheitsaktivität der rheumatischen Erkrankung als krankheitsspezifische Risikofaktoren für die Notwendigkeit einer stationären Behandlung wegen COVID-19 identifiziert werden. Auswertungen der kontinuierlich wachsenden Kohorte von Patienten mit entzündlich rheumatischen Erkrankungen und einer COVID-19-Infektion erlauben, Handlungsempfehlungen für die Betreuung der Patienten auf eine bessere Evidenz zu stützen. Die Kooperation mit internationalen rheumatologischen Registern (z. B. europäisches COVID-19-Register für ERE) ermöglicht Analysen aggregierter Kohortendaten von Patienten mit entzündlich rheumatischen Erkrankungen und einer SARS-CoV-2-Infektion für internationale Vergleiche und statistisch noch besser abgesicherte Aussagen.

## Abstract (en)

The COVID-19 registry (www.covid19-rheuma.de) of the German Society of Rheumatology was the first registry for the acquisition and systemic evaluation of viral infections in patients with inflammatory rheumatic diseases (IRD). This has enabled rapid generation of scientific data that will help to improve the care of patients with IRD in the context of the pandemic. In addition to confirming general risk factors, such as patient age and comorbidities (e.g. cardiovascular, chronic lung and kidney diseases), the use of glucocorticoids and the disease activity of the rheumatic disease could be identified as disease-specific independent risk factors for the need of hospitalization due to COVID-19. Evaluations of the continuously growing cohort of patients with IRD and COVID-19 enable recommendations for patient care to be based on better evidence. Cooperation with international rheumatology registries (e.g. European COVID-19 registry for IRD) enables analyses of aggregated cohorts of patients with IRD and COVID-19 for international comparisons and statistically even more reliable statements.

Mit dem Ausbruch des Severe Acute Respiratory Syndrome Coronavirus 2 (SARS-CoV‑2 bzw. COVID-19) in Wuhan im Dezember 2019 entwickelte sich aus einem lokalen Infektionsgeschehen binnen 3 Monaten eine Pandemie [1]. Die Deutsche Gesellschaft für Rheumatologie (DGRh) veröffentlichte am 30.03.2020 erste, konsentierte Handlungsempfehlungen für die Betreuung^1^ mit entzündlich rheumatischen Erkrankungen (ERE) im Rahmen der COVID-19-Pandemie, die sich naturgemäß noch nicht auf Studien zu Infektionen mit SARS-CoV‑2 stützen konnten [2]. Basis hierfür waren Daten von bekannten viralen Infektionen der oberen und unteren Atemwege, z. B. mit Influenzaviren oder bekannten Coronaviren, wie SARS (schweres akutes respiratorisches Syndrom) oder MERS (Middle East Respiratory Syndrome), welche mit einer COVID-19 vergleichbaren initialen Klinik (Husten, Fieber, Zephalgien und Myalgien), demselben Übertragungsweg (Tröpfcheninfektion) und variabler Symptomatik, von symptomlos bis zu kurzen letalen Verläufen, einhergehen. Im Hinblick auf die immunmodulierende Therapie wurde von einem Pausieren oder einer Reduktion aus Sorge vor einer Infektion ausdrücklich abgeraten, da man – wiederum in Analogie zu bekannten Infektionen bei ERE – davon ausging, dass dadurch Krankheitsschübe begünstigt werden, die auch das Risiko für eine SARS-CoV-2-Infektion erhöhen dürften, spätestens wenn man zur Behandlung eines Schubes wieder höhere Glukokortikoiddosen (GC) einsetzen müsste [2]. Die Fortsetzung der immunmodulierenden Therapie setzt neben einer adäquaten und engmaschigen rheumatologischen Betreuung mit enger Arzt-Patienten-Kommunikation, auch eine entsprechende Compliance der Patienten voraus. In einer longitudinalen Befragung von Patienten aus rheumatologischen Ambulanzen und Praxen in Deutschland über einen Zeitraum von 3 Monaten konnte gezeigt werden, dass die Mehrheit der Patienten angab, ihre Therapie gemäß der DGRh-Empfehlung fortzuführen [3].

Um Erkenntnisse zum adäquaten Umgang mit der immunmodulatorischen Therapie bei Patienten mit ERE im Kontext der COVID-19-Pandemie zu gewinnen, sind Registerdaten mit einer hohen Fallzahl sehr hilfreich. Diese erlauben, das Risiko für einen schweren Verlauf einer SARS-CoV-2-Infektion bei Patienten mit verschiedenen ERE oder unter einer bestimmten Therapie besser abzuschätzen. Aus diesem Grund initiierte die DGRh bereits im März 2020 gemeinsam mit der Justus-Liebig-Universität Gießen ein Online-Register (www.covid19-rheuma.de), mit dessen Hilfe nachgewiesene SARS-CoV-2-Infektionen (positiver PCR- oder Antikörpertest) bei Patienten mit ERE innerhalb weniger Minuten erfasst werden können. Im Register werden u. a. folgende Aspekte erfasst: Bundesland, Alter, Geschlecht, Gewicht, Größe, Impfstatus (Grippe‑, Pneumokokken- und SARS-CoV-2-Impfung), Komorbiditäten, Krankheitsaktivität und Therapie der rheumatischen Grunderkrankung zum Zeitpunkt der SARS-CoV-2-Infektion und deren Verlauf. Dieses bundesdeutsche Register soll auch dazu dienen, den Verlauf oder den Ausgang von COVID-19 bei Patienten mit ERE in Deutschland mit anderen Ländern vergleichen zu können, die sich z. B. im Hinblick auf die medizinische Versorgung und das jeweilige Gesundheitssystem unterscheiden [4].

## Vernetzung des COVID-19-Rheuma Registers

Bereits bei der Konzeption des COVID19-Rheuma.de Registers wurde darauf geachtet, Inhalte und Datenbankstruktur so zu gestalten, dass die Daten auch mit anderen nationalen und internationalen Registern vergleichend ausgewertet werden können. Die Daten des deutschen Registers können so auch in das europäische Register (EULAR-COVID-19-Registry) sowie in das internationale Register (COVID-19-Global Rheumatology Alliance) übertragen werden, sodass keine Doppeleingabe der deutschen Patienten in die internationalen Register nötig ist. Aus dieser Zusammenarbeit resultierte bereits eine weitere, wichtige Publikation zu Faktoren, die mit einer verstärkten Hospitalisierung bei COVID-19 und ERE assoziiert sind [5]. Neben der Verbindung zu den anderen rheumatologischen COVID-19-Registern wurde eine Kooperation zwischen dem DGRh-Register und dem Lean European Open Survey on SARS-CoV‑2 infected patients(LEOSS)-Register aufgebaut, welches SARS-CoV-2-Infektionen insgesamt europaweit erfasst [6]. Auf dieser Kooperation basierend, ist aktuell eine Analyse des Verlaufes einer SARS-CoV-2-Infektion bei rheumatologischen Patienten im Vergleich zu Patienten ohne ERE oder anderen Autoimmun‑/Tumorerkrankungen geplant.

## Analysen aus dem COVID-19-Rheuma Register im ersten Jahr der Pandemie

Im Rahmen der ersten Publikation zum COVID-19-Register erfolgten die Beschreibung des Aufbaus des DGRh-Registers sowie eine erste Analyse der Akzeptanz und Rekrutierung nach einem Dokumentationszeitraum von 4 Wochen [7]. In diesem Zeitraum, welcher v. a. die erste Welle der COVID-19-Pandemie in Deutschland umfasste, wurden 104 Patienten erfolgreich dokumentiert, und der Einsatz von GC kristallisierte sich als ein erster möglicher Risikofaktor für eine Hospitalisierung im Rahmen von COVID-19 heraus [7].

In der Folgepublikation mit zum damaligen Zeitpunkt 468 erfassten Patienten konnten Risikofaktoren hinsichtlich einer Hospitalisierung bei einer SARS-CoV-2-Infektion sehr viel genauer analysiert werden [8]. Hierbei wurden Patientenalter, kardiovaskuläre Komorbiditäten, chronisch interstitielle Lungenerkrankungen bzw. chronisch obstruktive Lungenerkrankungen und der Einsatz von GC als unabhängige Faktoren für die Notwendigkeit einer stationären Behandlung von COVID-19 bei Patienten mit ERE identifiziert. Besonders hervorzuheben war, dass auch die Krankheitsaktivität der ERE als ein unabhängiger Prädiktor für eine Hospitalisierung identifiziert werden konnte [8]. Dies zeigte sich auch in der Analyse der globalen Registerdaten, bei der eine erhöhte Krankheitsaktivität signifikant assoziiert war mit COVID-19-bedingter Mortalität [9]. Hierdurch ließ sich die Empfehlung der DGRh, bestätigen, dass eine adäquat immunmodulatorisch therapierte ERE ein geringeres Risiko für eine Hospitalisierung bzw. einen schweren Krankheitsverlauf bei einer SARS-CoV-2-Infektion haben dürfte [8, 9]. Eine rasch zunehmende Zahl von Publikationen zu COVID-19 generell und speziell bei Patienten mit ERE, hier insbesondere auch aus den Registern, war für die DGRh dann auch Anlass, die ersten Handlungsempfehlungen nach systematischer Literaturrecherche bereits Mitte 2020 zu aktualisieren [10].

## Aktueller Stand des Registers

**Figure 1:**
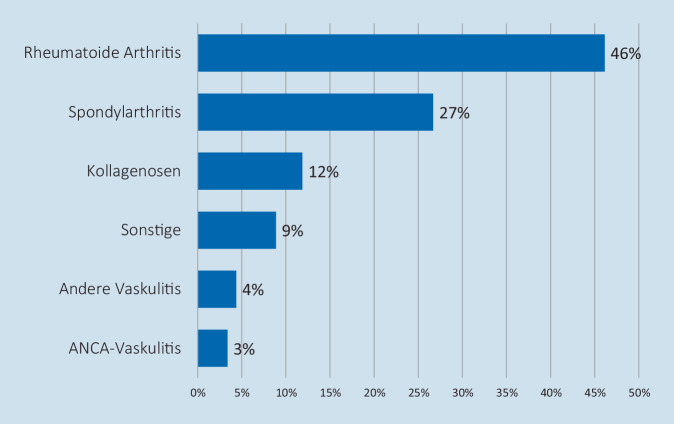

**Figure 2:**
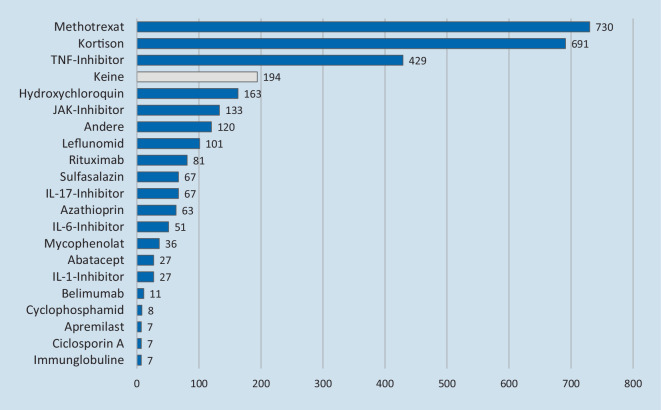

**Figure 3:**
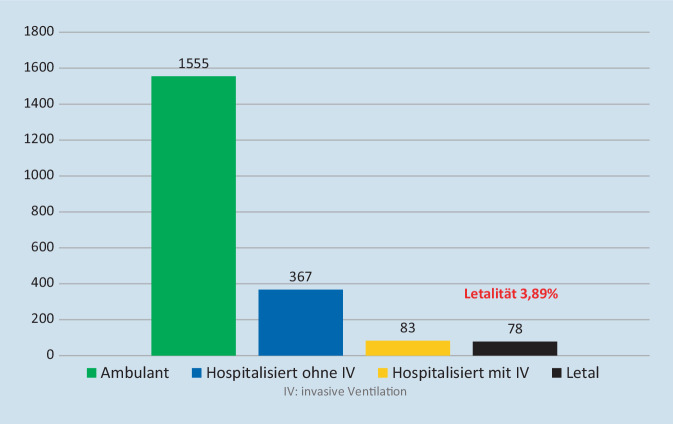

Aktuell sind im Register 2005 Patienten erfasst (Stand: 21.03.2021). Mehrheitlich stammen die eingeschlossenen Patienten aus den Bundesländern Bayern, Baden-Württemberg, Nordrhein-Westfalen und Hessen. Diejenigen Bundesländer, die in der Allgemeinbevölkerung höhere Zahlen von SARS-CoV-2-Infektionen aufweisen, sind im rheumatologischen Register ebenfalls stärker vertreten (Tab. 1). Das mittlere Alter der 1348 Frauen, 655 Männer und 2 diversgeschlechtliche Personen beträgt 65 Jahre (Range 19 bis 96 Jahre). Die Haupterkrankungsbilder stellen die rheumatoide Arthritis (RA, 46 %), die Spondyloarthritiden (SpA, 27 %) einschließlich der Psoriasisarthritis und die Kollagenosen (12 %) dar. Des Weiteren weisen 3 % der eingeschlossenen Patienten eine ANCA(antineutrophile zytoplasmatische Antikörper)-assoziierte Vaskulitis auf (Abb. 1). Mehrheitlich wurden die Patienten mit Methotrexat therapiert, gefolgt von GC und TNF-Inhibitoren (Abb. 2). Positiv zu vermerken ist, dass aktuell 1630 Patienten bereits genesen sind, allerdings wurden auch 78 letale Verläufe gemeldet, die mehrheitlich in der zweiten Welle der Pandemie erfasst wurden (Abb. 3).

**Table 1:** 

	DGRh COVID-19-Register	COVID-19-Fälle in der Allgemeinbevölkerung (laut Robert Koch-Institut)
Bayern	412 (21 %)	472.427 (18 %)
Nordrhein-Westfalen	262 (13 %)	577.480 (22 %)
Hessen	241 (12 %)	205.734 (8 %)
Baden-Württemberg	239 (12 %)	343.165 (13 %)
Berlin	215 (11 %)	138.205 (5 %)
Sachsen	148 (7 %)	209.156 (8 %)
Brandenburg	116 (6 %)	82.945 (3 %)
Rheinland-Pfalz	82 (4 %)	109.639 (4 %)
Hamburg	71 (4 %)	56.992 (2 %)
Niedersachsen	69 (3 %)	183.678 (7 %)
Saarland	66 (3 %)	30.668 (1 %)
Schleswig-Holstein	39 (2 %)	46.969 (2 %)
Sachsen-Anhalt	18 (< 1 %)	67.601 (3 %)
Thüringen	18 (< 1 %)	87.286 (3 %)
Mecklenburg-Vorpommern	7 (< 1 %)	27.936 (1 %)
Bremen	2 (< 1 %)	19.635 (1 %)
*Gesamt*	*2005*	*2.659.516*

## Aktuelle Publikationen und weitere Projekte

Zur Frage, inwieweit sich der Verlauf einer SARS-CoV-2-Infektion bei verschiedenen rheumatologischen Diagnosen unterscheidet, erfolgte in der aktuellsten Publikation aus dem Register eine erste Analyse der beiden größten Krankheitsgruppen: rheumatoide Arthritis (RA) und Spondyloarthritis (SpA) [11]. Hierbei wies die Gruppe der SpA eine niedrigere Hospitalisierungsrate (16 % vs. 30 %) auf. Dies könnte durch den geringeren Einsatz von GC bedingt sein, welche lediglich 13 % der SpA-Patienten erhielten gegenüber 40 % der RA-Patienten. Bezüglich der letalen Verläufe zeigte sich aber kein signifikanter Unterschied [11].

Flankierend wurde zu dem deutschen COVID-19-Register eine Online-Umfrage zu Auswirkungen der Corona-Pandemie auf Rheumapatientinnen und -patienten aufgesetzt, welche prospektiv über 1 Jahr versucht, die rheumatologische Versorgung und den Umgang der Patienten mit den Problemen, welche die Pandemie für diese mit sich bringt, zu erfassen. Zwischen April und Juli 2020 hatten sich insgesamt 695 Patienten eingeschrieben, sodass dieses Projekt im August 2021 ausgewertet werden kann.

Des Weiteren erfolgte im Februar und März 2021 eine Umfrage unter deutschen Rheumatologen, um deren Einstellung zur Corona-Impfung und zu dem Umgang mit Fragen ihrer Patienten zur Impfproblematik zu analysieren. Die Antworten von insgesamt 214 Ärztinnen und Ärzten werden derzeit ausgewertet.

Im Rahmen der aktuellen Aktivität der Ad-hoc-Kommission COVID-19 der DGRh erfolgt eine Erfassung von Corona-Impfungen bei Patienten mit ERE in einem separaten Impfregister. Darin werden Patienten mit ERE gebeten, Fragen zu Verträglichkeit, Sicherheit und Effektivität der COVID-19-Vakzinierung über einen Zeitraum von 12 Monaten in mehrfachen kurzen Online-Befragungen zu beantworten. Dieses Impfregister wurde Anfang Februar 2021 gestartet und umfasst bislang bereits 106 Patienten (Stand: 21.03.2021). Für Patienten, die nicht selbst an der Online-Impferfassung teilnehmen können, besteht die Möglichkeit einer papierbasierten Erhebung (Download des Formulars unter www.covid19-rheuma.de, Infobox 1).

### Infobox 1 Zugang zum Register und zur Impferfassung für Patienten


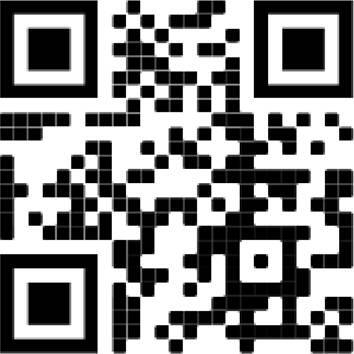
Zugang zum Register:www.covid19-rheuma.de


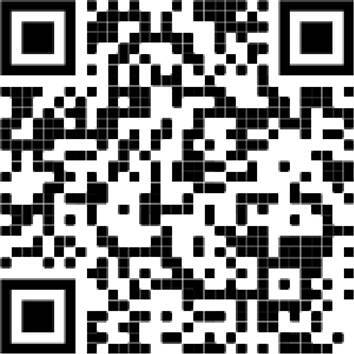
Zugang zur Impferfassung für Patienten:https://www.covid19-rheuma.de/patienten-information-impfung


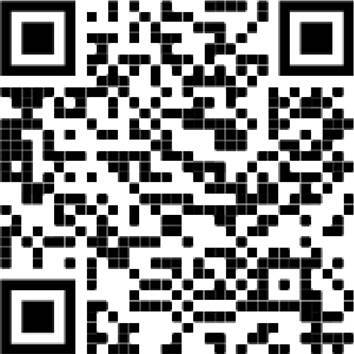
Zugang zur Papierversion der Impferfassung:https://www.covid19-rheuma.de/pdf/fragebogen-impfung-20210413.pdf

## Abkürzungen

COVID-19: Corona Virus Disease 2019
DGRh: Deutsche Gesellschaft für Rheumatologie
GC: Glukokortikoide
LEOSS: Lean European Open Survey on SARS-CoV‑2 infected patients
RA: Rheumatoide Arthritis
SARS-CoV‑2: Severe Acute Respiratory Syndrome Coronavirus 2
SpA: Spondyloarthritis

## Footnotes

1 In dieser Arbeit wird aus Gründen der besseren Lesbarkeit das generische Maskulinum verwendet. Weibliche und anderweitige Geschlechteridentitäten werden dabei ausdrücklich mitgemeint, soweit es für die Aussage erforderlich ist.
